# MRI-based measurements of aerosol deposition in the lung of healthy and elastase-treated rats

**DOI:** 10.1152/japplphysiol.01165.2013

**Published:** 2014-05-01

**Authors:** Jessica M. Oakes, Ellen C. Breen, Miriam Scadeng, Ghislain S. Tchantchou, Chantal Darquenne

**Affiliations:** ^1^Department of Mechanical and Aerospace Engineering, University of California, San Diego, California;; ^2^Department of Medicine, Division of Physiology, University of California, San Diego, California;; ^3^Department of Radiology, University of California, San Diego, California; and; ^4^Department of Chemical Engineering, University of California, San Diego, California

**Keywords:** particle, emphysema, heterogeneity, spatial distribution

## Abstract

Aerosolized drugs are increasingly being used to treat chronic lung diseases or to deliver therapeutics systemically through the lung. The influence of disease, such as emphysema, on particle deposition is not fully understood. With the use of magnetic resonance imaging (MRI), the deposition pattern of iron oxide particles with a mass median aerodynamic diameter of 1.2 μm was assessed in the lungs of healthy and elastase-treated rats. Tracheostomized rats were ventilated with particles, at a tidal volume of 2.2 ml, and a breathing frequency of 80 breaths/min. Maximum airway pressure was significantly lower in the elastase-treated (P_aw_ = 7.71 ± 1.68 cmH_2_O) than in the healthy rats (P_aw_ = 10.43 ± 1.02 cmH_2_O; *P* < 0.01). This is consistent with an increase in compliance characteristic of an emphysema-like lung structure. Following exposure, lungs were perfusion fixed and imaged in a 3T MR scanner. Particle concentration in the different lobes was determined based on a relationship with the MR signal decay rate, R_2_^*^. Whole lung particle deposition was significantly higher in the elastase-treated rats (C_E,part_ = 3.03 ± 0.61 μm/ml) compared with the healthy rats (C_H,part_ = 1.84 ± 0.35 μm/ml; *P* < 0.01). However, when particle deposition in each lobe was normalized by total deposition in the lung, there was no difference between the experimental groups. However, the relative dispersion [RD = standard deviation/mean] of R_2_^*^ was significantly higher in the elastase-treated rats (RD_E_ = 0.32 ± 0.02) compared with the healthy rats (RD_H_ = 0.25 ± 0.02; *P* < 0.01). These data show that particle deposition is higher and more heterogeneously distributed in emphysematous lungs compared with healthy lungs.

chronic lung diseases, such as asthma (21), chronic obstructive pulmonary disease (COPD), and cystic fibrosis (8) are increasingly being treated with aerosolized therapeutics. Additionally, the lung is gradually being used as a portal of entry for inhalable drugs to treat systemic diseases, e.g., insulin for diabetic patients (22). Therefore, there is a need to understand the means by which these drugs can most effectively be targeted to desired regions of the lung while avoiding delivery to other regions. Lung diseases are often accompanied by airway narrowing, change in lung compliance, and/or mucus plugging. These factors highly influence the distribution of inhaled drugs and thus their deposition pattern. Emphysema is characterized by alveolar wall destruction (18), air space enlargement, a decrease in small airway diameter (10), and an increase in tissue compliance.

While detailed, yet noninvasive, studies of peripheral aerosol deposition in humans have advanced in recent years (9, 17), there is still a need to use both computational and animal models. This is particularly true for studying emphysema, as very little work has been done to study the influence of emphysema-dependent changes in lung structure on particle deposition in human subjects. Also, studies of aerosol deposition in patients with obstructive lung diseases have had mixed outcomes. Brand et al. (3) found no difference in particle deposition between healthy, emphysematous, and cystic fibrosis patients for 4-μm-diameter particles. Kim and Kang (7) showed increased deposition of 1-μm-diameter particles in patients with obstructive lung disease compared with healthy controls. Several authors have also used animal models to study the effect of emphysema on aerosol deposition. For example, Sweeney et al. (16) found the deposition of 0.45-μm-diameter particles to be less, but more heterogeneously distributed, in elastase-treated hamsters compared with healthy hamsters. The advantage of using rodent models is that typically more spatial information can be gained by using invasive techniques. Also, as much of the assessment of pharmacological and toxicological effects of inhaled particles still comes from studies in small rodents, information about deposition of aerosol particles in the respiratory tract of these animals is required for meaningful interpretation of toxicological studies.

We have recently developed a magnetic resonance imaging (MRI)-based method, which uses the change in signal decay rate (R_2_^*^), due to the presence of iron oxide particles, to quantify the spatial distribution of deposited particles in rodent lungs (13). We showed that R_2_^*^ was higher in rat lungs exposed to iron-oxide particles, compared with the non-aerosol-exposed lungs and that this difference in R_2_^*^ reflected the presence of deposited particles in lung tissue. By imaging a phantom with known particle concentrations, we showed that R_2_^*^ is proportional to the concentration of particles. Our previous study used healthy rats to demonstrate the feasibility of using MRI to detect and quantify regional aerosol particle deposition in rodent lungs. However, this method can be readily applied to a variety of animal models of human disease.

The goal of the current study was to determine the effect of emphysema-like lung morphology on aerosol deposition in the rat lung. Specifically, we determined the effect of elastase treatment on the spatial distribution of deposited particles. Iron oxide particles with a mass median aerodynamic diameter (MMAD) of 1.2 μm were delivered in a controlled manner to the lungs of healthy and elastase-treated rats before being imaged ex vivo in a 3T MR scanner (13). The concentration and heterogeneity of the deposited particles were determined in each lobe of the elastase-treated and healthy rat lungs. Additionally, particle deposition in the central and peripheral regions was determined. This study is the first to determine changes in deposition between healthy and elastase-treated rat lungs exposed to aerosolized particles with similar breathing patterns and may be used to validate numerical simulations of particle transport in the rat lung (11).

## METHODS

### Elastase Induction

The study protocol was approved by the University of California, San Diego Institutional Animal Care and Use Committee. Emphysema-like morphology was induced in 13 healthy male Wistar rats (6 wk old, body wt = 195 ± 12 g). The animals were anesthetized with isoflurane before being orotracheally instilled with porcine pancreatic elastase (Sigma Aldrich; 125 U/kg body wt) diluted in 0.5 ml of saline. After instillation, the rats were gently rocked to encourage homogeneous enzyme exposure until they regained consciousness. The rats were administered 100% oxygen through a plastic cannula directed at the animal nares throughout the procedure. The rats were then kept in an oxygenated chamber warmed to 37°C for 15 min to aid in recovery before being returned to their cages. Animals were housed in the vivarium and monitored daily. They were provided food and water ad libitum and were on 12-h lights on and 12-h lights off cycle. The 13 elastase-treated (E) rats (13.5 wk old, body wt = 420 ± 39 g) were studied 6 wk later along with 11 healthy (H) weight-matched rats (15 wk old, body wt = 402 ± 23 g).

### Aerosol Exposure

The aerosol exposure protocol was similar to that previously described in Oakes et al. (13). Briefly, anesthetized (1.25 mg xylazine/8.75 mg ketamine per 100 g body wt), tracheostomized rats were mechanically ventilated (breathing frequency: 80 breaths/min; tidal volume: 2.2 ml; positive end-expiratory pressure: 1 cmH_2_O; Harvard Apparatus Model No. 683) for 40 min with either particle-free air (H: *n* = 5; E: *n* = 6) or particle-laden air (H: *n* = 6; E: *n* = 7). Particle concentration was ∼5,000 particles/ml of air (13). Time varying airway pressure was continuously monitored throughout the exposure by a pressure transducer connected to the tracheal cannula. The aerosol was made of monodisperse magnetic polystyrene particles with a geometric diameter of 0.95 μm and a density of 1.35 g/cm^3^ (Kisher Biotechnologies) suspended in water at a 1:4 ratio. The MMAD of the particles was 1.2 μm.

After the exposure period, lungs were perfused through the vasculature with saline followed by 3% glutaraldehyde mixed in 0.01 M phosphate buffer at an airway pressure of 20 cmH_2_O and vascular pressure of 15 cmH_2_O for 20 min (11). Fixation was performed through the vasculature rather than by tracheal instillation to minimize the risk of particles being dislocated during the fixation process. Lungs were then excised and stored in MR-compatible containers (3 lungs per container) filled with fixative for 3 mo before being degassed under a light vacuum for ∼2 wk so that the lungs were filled with liquid for the imaging session.

### MRI and Image Processing

**Fig. 1.**
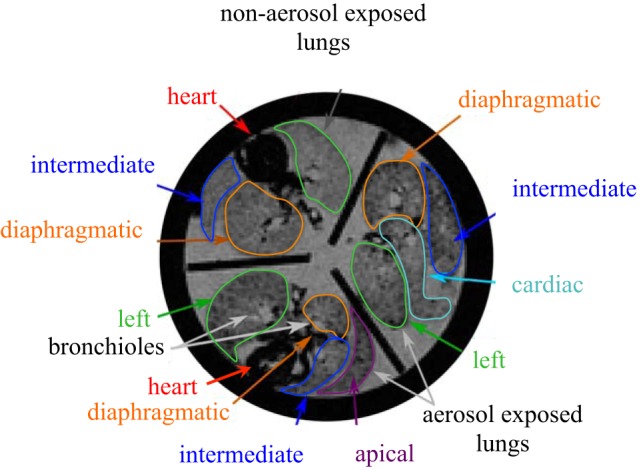
Representative magnetic resonance (MR) signal intensity image (echo time of 100 ms) of 1 control and 2 aerosol-exposed healthy lungs with the 5 lobes identified.

The imaging protocol was the same as described previously (13). Briefly, the lungs were held in a polycarbonate MR-compatible container, which was placed in the center of a large cylindrical plastic vessel filled with water. The large vessel of water reduced the field inhomogeneities that would otherwise be present at the interface of the MR container and air (13). Images were collected with a 3T General Electric 750 MR scanner using a GE 18-cm-diameter transmit and receive knee coil. A gradient echo imaging sequence was used with a flip angle of 20°, a repetition time of 2 s, a field of view of 13 cm, and echo times of 8.2, 40, 100, and 200 ms. Approximately 32 transaxial images were obtained with an in-plane resolution of 500 μm and thickness of 1 mm. Figure 1 shows a representative transaxial MR image of the MR-compatible container housing one control and two aerosol-exposed healthy lungs.

The signal decay rate, R_2_^*^, was calculated on a per-voxel basis for each original signal intensity axial image (13). The coefficient of variation, *R*^2^, was determined for each R_2_^*^ calculation and only values with *R*^2^ > 0.75 were used in the analysis, resulting in removal of ∼5% of the voxels from the study. Regions of interest (ROI) were hand drawn around each lobe of the lung. Each of the rat lobes [left lung: left lobe; right lung: apical, intermediate, diaphragmatic, and cardiac lobes (12)] were identified from the images as shown in Fig. 1. Additionally, ROIs were drawn around the central and peripheral regions of each lobe, as described previously (13). The heterogeneity of the signal decay rate, R_2_^*^, in each lobe was assessed by the RD [RD = standard deviation (SD)/mean].

### Particle Concentration

Particle concentration C_part_(*i*,*j*,*k*) was calculated on a per voxel basis by using a linear relationship previously determined from a calibration phantom made of agarose and fixative that contained known particle concentrations (13)

(1) $${\text{C}}_{\text{part}}(i,j,k)=\frac{R_{2}^{*}(i,j,k)-R_{2,\text{control}}^{*}(k)}{m}$$

where (*i*,*j*,*k*) defines the position of the voxel in the x and y direction and the transaxial position, respectively; R_2_^*^(*i*,*j*,*k*) is the voxel signal decay rate; $R_{\mathrm{2,control(k)}}^{*}$ is the mean R_2_^*^ for the control, non-aerosol-exposed lungs; and *m* is the slope derived from the particle concentration calibration experiment, *m* = 0.00107 μg·ml^−1^·ms^−1^ (13). The mean $R_{\mathrm{2,control(k)}}^{*}$ that was used to calculate C_part_(*i*,*j*,*k*) was the mean R_2_^*^ of each lobe and lung region (central or peripheral) for each transaxial position of the corresponding healthy or elastase-treated control lungs. Negative particle concentration values were set to zero. Negative particle concentrations were likely caused by a combination of low lung tissue density [well below the average $R_{\mathrm{2,control(k),}}^{*}$ *Eq. 1*] and particle concentration below the detectible limit. The total number of particles depositing in each lobe was determined by multiplying the particle concentration by the lobe volume. This value was normalized by the total number of particles depositing in the lung and was defined as the normalized particle deposition. The volume-normalized deposition was found by dividing the normalized particle concentration by the volume fraction, where the volume fraction was the volume of each lobe divided by the total lung volume.

### Alveolar Morphometry

Post-MRI, seven healthy and seven elastase-treated lungs were randomly chosen for morphometric analysis of air space size. Lung lobes were embedded in paraffin, sectioned into 7-μm thick slices, and stained with Harris modified hematoylin (Fisher Scientific) (19). The slides were imaged with a NanoZoomer 2.0 HT Slide Scanning System at a magnification of 20 (resolution 7.25 × 7.25 μm).

The mean linear intercept (L_M_) was calculated from scanned images using an in-house Matlab code. Briefly, the grey scale images were turned into binary images with black voxels (alveolar tissue) and white voxels (air spaces). Vertical lines spaced 72.5 μm apart were drawn on a ROI in each image, avoiding large airways. The number of times white voxels turned to black on each vertical line, i.e., each time the line crossed an alveolar or duct wall, was calculated. These measurements were averaged over all vertical lines in the ROI. The mean linear intercept were then calculated as the length of the vertical line divided by the number of times the line intersected with alveolar septa. To check for accuracy, this method was validated against manually counting the number of crossings for one image.

### Statistical Analysis

**Fig. 2.**
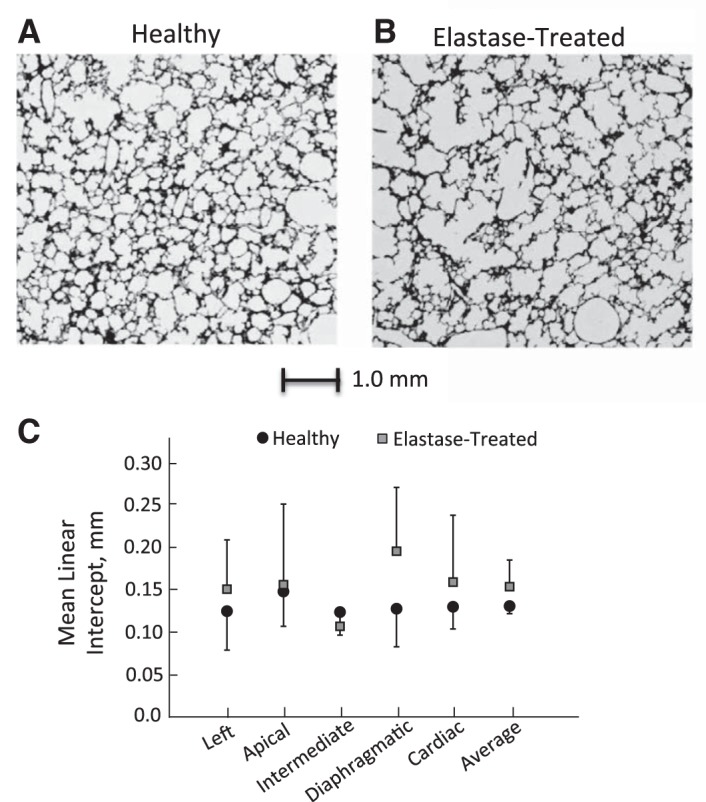
Representative morphometric images for healthy (*A*) and elastase-treated (*B*) rats. *C*: averaged mean linear intercept (L_M_) for each lobe and for the lung as a whole (i.e., average) with the error bars representing the SD between rats.

**Fig. 3.**
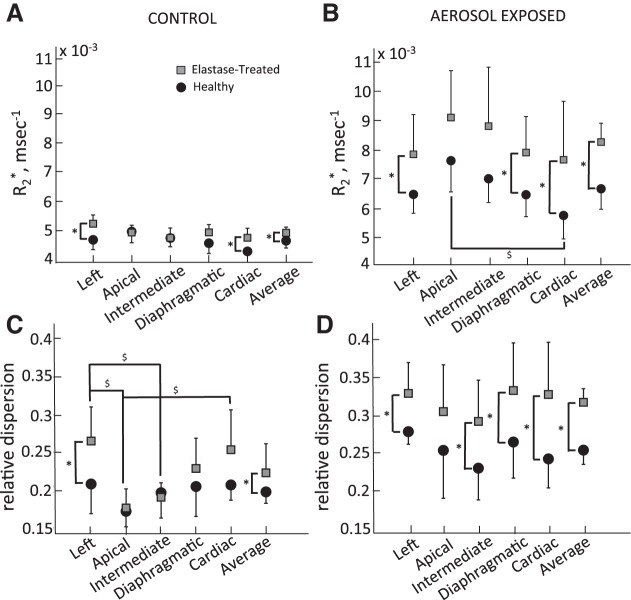
Signal decay rate (R_2_^*^) and its relative dispersion (RD) in the control (*A* and *C*) and aerosol-exposed animals (*B* and *D*). In both the control (no exposure) and aerosol-exposed groups, R_2_^*^ and RD were significantly higher in the elastase-treated rats than in the healthy rats (*P* = 0.003 and *P* = 0.012, respectively, for control animals and *P* < 0.001 for both R_2_^*^ and RD in aerosol-exposed animals). *Statistical significance between diseased and healthy rats for a given lobe. $Statistical significance between lobes within each disease category.

**Fig. 4.**
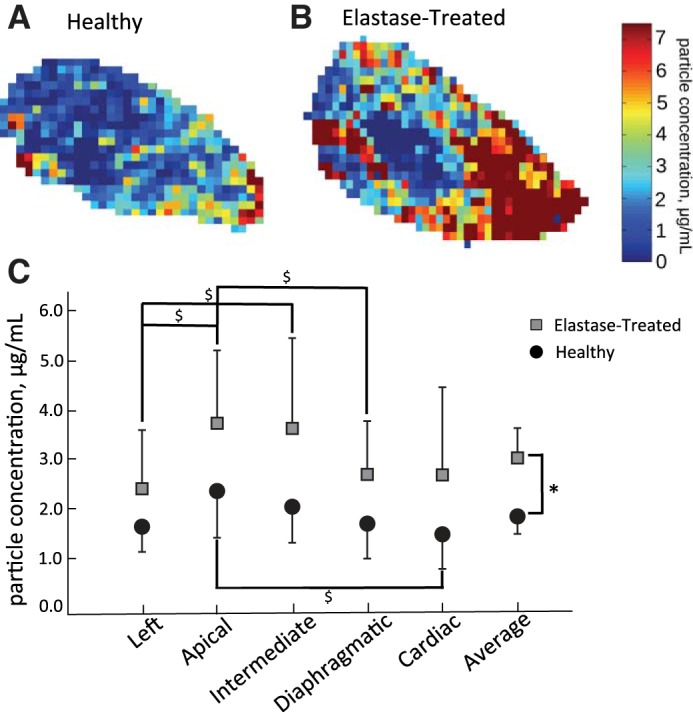
Concentration of deposited particles. *A* and *B*: representative particle concentration maps for healthy and elastase-treated rats, respectively. *C*: average particle concentration in each lobe of the healthy and elastase-treated rat lungs. Error bars represent the SD between rats and * denotes statistically significant difference between the healthy and elastase-treated rats (p < 0.01). No statistical significance was found when comparing the particle concentration in the individual lobes of the healthy and elastase-treated aerosol-exposed rats. $*P* < 0.05, statistical significance between lobes in each disease category.

**Fig. 5.**
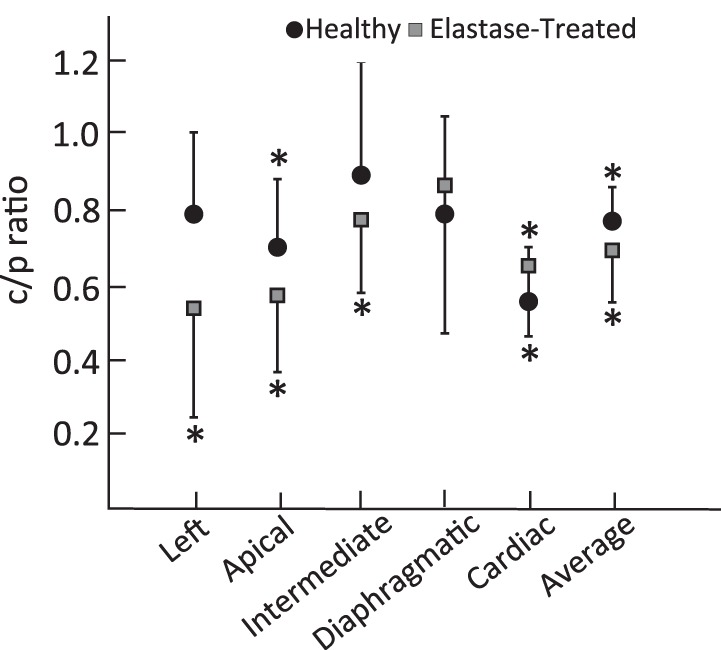
Central-to-peripheral ratio (C/P) in the healthy and elastase-treated aerosol-exposed rats. Error bars indicate SD between rats in each category. *P < 0.05, statistically different than 1.

**Fig. 6.**
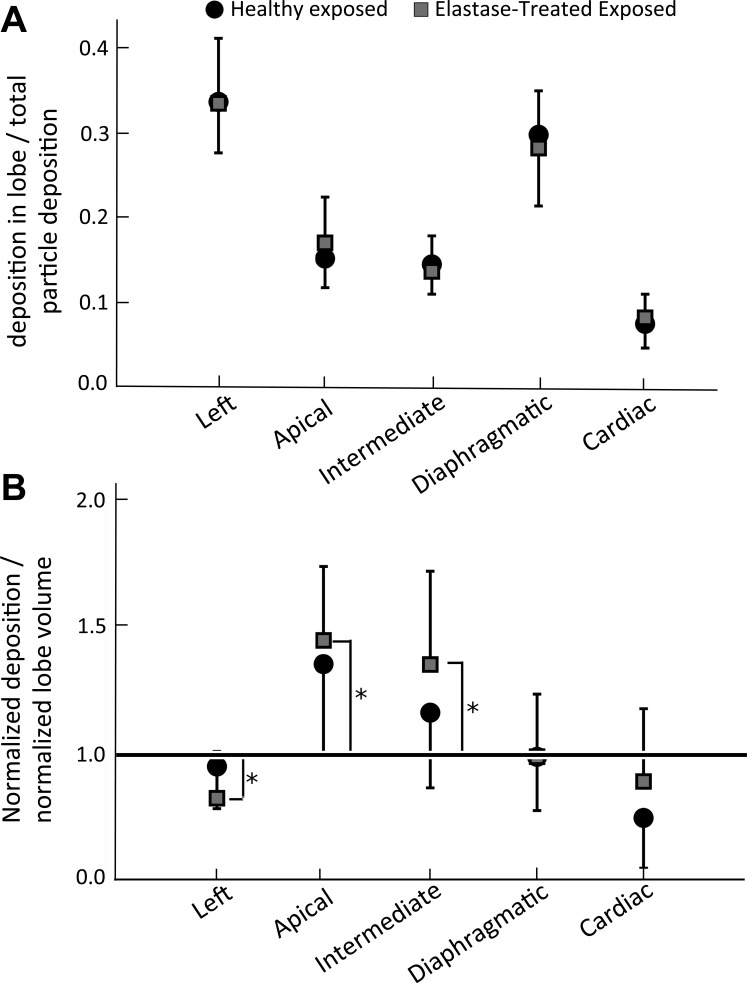
*A*: lobar deposition normalized by total lung deposition (normalized deposition). *B*: shows this normalized deposition divided by the lobe volume; a value of 1 would indicate that particle deposition in a given lobe is directly proportional to the lobe volume. Error bars represent 1 SD between rats. *Volume normalized deposition is statistically different than 1, p < 0.05.

All statistical analyses were performed with Systat version 11 (Systat, Evanston, IL). Data were grouped in different categorical variables: disease (emphysema and healthy), lung region (lobes or central and peripheral), and animal number. A two-way ANOVA was performed to test for differences in R_2_^*^ and RD between healthy and elastase-treated rats (see Fig. 3), and a one-way ANOVA was performed to test for differences in C_part_, normalized particle concentration, and mean linear intercept between groups (see Figs. 2, 4, and 6*A*). Post hoc testing using the Bonferroni adjustment was performed for tests showing significant *F*-ratios. Finally, a standard *t*-test was used to test for differences in the airway pressure between animal groups and to test if the central to peripheral (C/P) ratio and volume-normalized concentration were different from 1 (see Figs. 5 and 6*B*). Significant differences were accepted at the *P* < 0.05 level.

## RESULTS

### Alveolar Morphometry and Evidence of Emphysema

The maximum airway pressure (P_aw_) was significantly less in the elastase-treated rats (P_aw,E_ = 7.71 ± 1.68 cmH_2_O) compared with the healthy rats (P_aw,H_ = 10.43 ± 1.02 cmH_2_O; *P* < 0.01). Lobe volumes were measured from the MR images and the percentages of total lung volumes were the same as found previously (12). No difference in lobe volume and total lung volume were found between the healthy and emphysematous rats. Figure 2 shows representative histological sections from the left lung of a healthy (*A*) and elastase-treated (*B*) rat. Larger air spaces can be readily seen in the parenchyma of the elastase-treated lung compared with the healthy lung. The mean linear intercept (L_M_) of each lobe of the healthy and elastase-treated rats is shown in Fig. 2*C*. Overall, L_M_ was larger in the elastase-treated rats than in the healthy rats. However, this increase was not statistically significant (*P* = 0.17). The greatest change was found for the diaphragmatic lobe (*P* = 0.064) and would have most likely been significant if more lungs were analyzed. There were no differences in SD or RD of L_M_ between the healthy and elastase-treated rats.

### Signal Decay Rate and Its RD in Healthy and Elastase-Treated Lungs

Figure 3 displays R_2_^*^ and its RD in the healthy and elastase-treated rats for the control and aerosol-exposed groups. Data are shown as the means ± SD for each experimental group. Overall, R_2_^*^ (Fig. 3, *A* and *B*) and RD (Fig. 3, *C* and *D*) were significantly higher in elastase-treated rats than in healthy rats in both the control (air-exposed) and aerosol-exposed groups (*P* ≤ 0.012). Also, R_2_^*^ and RD were significantly higher in each aerosol-exposed group compared with their respective control (particle free air) group (*P* < 0.001).

#### Control (particle-free air-exposed) groups.

Mean R_2_^*^ values did not vary significantly between lobes in either the elastase-treated or the healthy group within each category (Fig. 3*A*). Comparing experimental groups, R_2_^*^ was significantly higher in the left (*P* = 0.018) and the cardiac (*P* = 0.041) lobes of the elastase-treated rats compared with the corresponding lobes in the healthy control group. While there was no significant difference in RD between lobes in the healthy control group, significant differences in RD were observed between lobes of the elastase-treated control group (apical vs. cardiac, *P* = 0.019; apical vs. left, *P* = 0.005; intermediate vs. left, *P* = 0.026; Fig. 3*C*). RD was also significantly higher in the left lobe of the elastase-treated group compared with that of the healthy group (*P* = 0.05; Fig. 3*C*).

#### Aerosol-exposed groups.

R_2_^*^ was significantly higher in the left, cardiac, and diaphragmatic lobes of the elastase-treated rats compared with the corresponding healthy lobes (*P* ≤ 0.05; Fig. 3*B*). For all lobes, RD was significantly higher in the elastase-treated group than in the healthy group (*P* ≤ 0.05) except for the apical lobe (Fig. 3*D*). There was no difference in R_2_^*^ and RD between lobes in either the aerosol-exposed elastase-treated or aerosol-exposed healthy group except for the apical lobe. R_2_^*^ in the apical lobe of the healthy group was significantly higher than R_2_^*^ in the cardiac lobe (Fig. 3*B*). RD in the whole lung was also higher (*P* < 0.01) in both the healthy (by 28.3%) and elastase-treated (by 42.5%) aerosol-exposed rats compared with their corresponding control rats. No change in R_2_^*^ along the axial axis was found for either the healthy or elastase-treated aerosol-exposed rats.

### Particle Concentration

Figure 4 shows particle concentration maps in a representative transaxial slice of the left lobe of a healthy (*A*) and elastase-treated (*B*) rat lung. The average particle concentration for each lobe of the healthy and elastase-treated animals are shown (Fig. 4*C*). In both groups (Fig. 4, *A* and *B*), particle concentration was heterogeneously distributed. Larger areas of high-particle concentration were observed in the elastase-treated group than in the healthy group. Within each group, particle concentration varied significantly between lobes, as shown in Fig. 4*C*. When averaged over the whole lung, particle concentration was significantly higher (*P* < 0.01) in the elastase-treated rats than in the healthy rats (Fig. 4*C*). However, the particle concentration was not statistically different between the respective lobes of the healthy and elastase-treated aerosol-exposed rats.

For both the elastase-treated and healthy groups, regional difference between the central and peripheral region was assessed in each lobe with the C/P ratio (Fig. 5). C/P was significantly <1, indicating a higher peripheral than central concentration, in the apical and cardiac lobes of the healthy rats and in all but the diaphragmatic lobe of the elastase-treated rats.

Finally, for each rat, the mass of particles in each lobe (the particle concentration in each lobe multiplied by the lobe volume) was normalized by the total particle mass in the whole lung (i.e., normalized particle deposition). Values averaged over all animals in both the aerosol-exposed healthy and elastase-treated rats are shown in Fig. 6*A*. There were no differences in the normalized particle deposition between the two groups. Figure 6*B* shows the volume-normalized particle deposition, the normalized particle deposition divided by the lobar volume fraction (lobe volume divided by total lung volume). A value of 1 would indicate that particle deposition is directly proportional to lobe volume. There were no differences between healthy and elastase-treated rats in the volume-normalized particle concentration (Fig. 6*B*). However, the left lobe was statistically <1 and the intermediate and apical lobes were statistically greater than 1 (Fig. 6*B*).

## DISCUSSION

The goal of the current study was to determine the effect of an emphysema-like morphology on aerosol deposition in the rat lung. The presence of an emphysema-like lung was evaluated by measuring maximum airway pressure during tidal breathing, the mean linear intercept (L_M_) and changes in R_2_^*^ in the control lungs. The concentration of deposited particles in each animal was spatially determined from the signal decay rate (R_2_^*^) in the aerosol-exposed lungs. This study is the first to present changes in particle deposition and distribution between healthy and elastase-treated rats exposed to aerosol particles under similar breathing patterns.

### Evidence of an Emphysema-Like Morphology

The extent of emphysema-like lung changes was determined by measuring the airway pressure (P_aw_) during mechanical ventilation and the mean linear intercept (L_M_) in all groups and R_2_^*^ in the control groups. For similar breathing patterns (tidal volume of 2.2 ml at 80 breaths/min), the maximum airway pressure was significantly lower in the elastase-treated rats than in the healthy rats. This is consistent with an increase in compliance that is characteristic of an emphysema-like lung structure. In a recent modeling study, Oakes et al. (11) calculated the respiratory compliance and resistance in the same group of rats used in this study. The tidal volume, inspiration time, and time varying tracheal pressure were used to solve a two compartment linear model (1). Respiratory compliance was significantly higher (*P* = 0.04) in the elastase-treated group (C_E_ = 0.37 ± 0.14 cm^3^/cmH_2_O) compared with the healthy group (C_E_ = 0.25 ± 0.04 cm^3^/cmH_2_O), while the resistance remained unchanged (*P* = 0.83). Although the compliances calculated by Oakes et al. (11) in the elastase-treated lungs were smaller than previously reported [C_E_ = 0.744 cm^3^/cmH_2_O (20); C_E_ = 0.92 ± 0.16 cm^3^/cmH_2_O (6)], the ratio between elastase-treated and healthy lung compliance (ratio of 1.4) was consistent with these previous studies [1.6 (20); 1.5 (6)].

Historically, the mean linear intercept (L_M_) has been used as an index for characterizing the severity of emphysema. While not statistically significant, our data showed a trend for L_M_ to be higher in the elastase-treated rats compared with the healthy rats with the greatest difference being observed in the diaphragmatic lobe (Fig. 2*C*). These data are similar to those of Emami et al. (5), who reported no significant change in L_M_ between Sprague-Dawley rats treated with elastase (20 IU/100 g body wt) and weight-matched healthy rats. It has been previously shown (14) that L_M_ may not be the best parameter to assess the severity of emphysema, especially in the presence of spatially heterogeneous tissue destruction. This may explain why statistical difference in L_M_ was not found between healthy and elastase-treated rats in the current study. This is discussed further in *Study Limitations*.

The impact of disease on the signal decay rate, R_2_^*^, was also explored by comparing R_2_^*^ in healthy and elastase-treated control rats (no aerosol exposure). In the control lungs, R_2_^*^ is essentially a measure of the presence of tissue, any residual blood, and microbubbles. Overall, and in the left and cardiac lobes, R_2_^*^ was significantly higher in the elastase-treated lungs compared with the healthy lungs. R_2_^*^ also tended to be higher in the diaphragmatic lobe of the elastase-treated rats compared with the healthy rats. Additionally, within the lobes, R_2_^*^ appeared to be higher at the base of the left, cardiac, and diaphragmatic lobes compared with the other regions of the lobe (data not shown). The higher R_2_^*^ in the elastase-treated lungs, compared with the healthy lungs, is likely due to either microscopic bubbles that were trapped and did not diffuse out of the lung during the degassing phase of the lung preparation protocol or due to residual blood that may not have been cleared as efficiently in the elastase-treated group during the saline perfusion procedure. As indicated by RD, the distribution of R_2_^*^ was more heterogeneously distributed in the elastase-treated rats compared with the healthy rats (Fig. 3*B*).

The increase in R_2_^*^ and RD in the elastase-treated rats compared with the healthy rats for the left, diaphragmatic, and cardiac lobes suggests that the disease was mainly present in these three lobes and did not develop as much in the intermediate and apical lobes. It is possible that since the elastase instillation was carried out through the trachea, the delivery rate to each lobe was not directly controlled. Therefore, it is likely that each lobe of the rat lung experienced a different level of tissue destruction. This preferential delivery may be due to structural features. Indeed, the average bifurcation angles leading to the apical and intermediate lobes are 48 ± 19° while the other three lobes branched at 22 ± 8°(12). It is possible that the instilled elastase traveled preferentially to the lobes with the smaller bifurcation angles, as it likely that the fluid momentum will cause it to continue in its initial direction. While several groups have used similar techniques to administer elastase in rodents (4, 6, 15), none of these studies reported the extent of tissue destruction in each lobe. Therefore, it is unknown if the areas of damage were homogeneously distributed in those previous studies or rather heterogeneously distributed as reported in this current study.

### Effect of Elastase Treatment on Particle Deposition

The presence of iron oxide particles in a tissue sample create local field inhomogeneities in the magnetic field, which result in an increase in the MR signal decay rate, R_2_^*^, compared with a particle-free tissue sample. This increase is directly proportional to the concentration of iron particles in the tissue sample (13). Even though there was a significant increase (*P* = 0.003) in R_2_^*^ between the elastase-treated and healthy lungs of the control (no particle) groups, this increase (5.7%; Fig. 3*A*) was less than the increase in R_2_^*^ between elastase-treated and healthy lungs of the aerosol-exposed groups (24%; Fig. 3*B*). This larger difference in R_2_^*^ between the aerosol-exposed lungs than between the control lungs resulted in a larger overall concentration of deposited particles in the elastase-treated group than in the healthy group (Fig. 4*C*). However, unlike for R_2_^*^, there was no significant difference in particle concentration when comparing the individual lobes of the two aerosol-exposed groups. Yet, there was a trend for deposition to be higher in the elastase-treated lungs than in the healthy animals as evidenced by *P* values ranging between 0.05 and 0.10 when comparing the individual lobes. Because of the rather large interanimal variability in deposition in the elastase-treated group (most likely because of the heterogeneous nature of the disease, Fig. 4*C*), a larger number of animals than that used in this study may be required to reach statistical significance.

In the control lungs, RD is a measure of the heterogeneity of lung structure. In the aerosol-exposed lungs, RD is a gauge of both the heterogeneity of lung structure and the heterogeneous distribution of deposited particles. Assuming the effects of heterogeneity of structure and heterogeneity of deposition are additive, the increase in RD between control and aerosol-exposed groups reflects the heterogeneity of deposition. The larger increase in RD between control and aerosol-exposed animals in the elastase-treated compared with the healthy group (42.5 vs. 28.3%) indicates that deposited particles are more heterogeneously distributed in the presence of emphysema-like morphology and tissue compliance, a result in agreement with previous observations made in hamsters by Sweeney et al. (16). Regional differences in tissue compliance will result in regional differences in rate and extent of lung expansion and contraction during breathing. In addition, the heterogeneous distribution of particle deposition may be linked to particle trapping caused by small airway collapse during exhalation in the emphysematous-like regions of the lung, while the particles in the healthy regions are exhaled normally.

In all lobes of the healthy and elastase-treated rats, particle concentration was higher in the peripheral region compared with the central region of the lung, as demonstrated by a C/P ratio <1 (Fig. 5). While the peripheral region mainly contains small and alveolated airways, the central region also includes the large and medium-sized conducting airways that account for ∼12 to 15% of the volume of the central region (12). Thus a C/P <1 strongly suggests that there was minimal deposition in the large and medium-sized airways, which is in agreement with a previous modeling study (11), where deposition was found to be <1% in the large airways for particles with MMAD of 1.2 μm.

Even though the overall concentration of deposited particles was higher in the elastase-treated lungs than in the healthy lungs, there was no significant difference in the relative lobar distribution of deposited particles between experimental groups (Fig. 6*A*). Interestingly, the volume-normalized deposition (Fig. 6*B*) was significantly >1 in the apical and intermediate lobes and significantly <1 in the left lobe. A value of 1 would indicate that particle deposition is directly proportional to lobe volume and would be true if ventilation is directly proportional to lobe volume and the rate of particle deposition is similar in each lobe. As the delivery of fresh air to a normal region is less than a diseased region (11), the >1 value in the apical and intermediate lobes may be reflective of enhanced deposition in the healthy regions of the lung compared with the diseased regions. Additionally, the smaller value of 1 in the left lobe may indicate lower deposition efficiency in this diseased lobe.

### Comparison with Previous Studies

There have been relatively few animal studies of particle deposition in emphysematous lungs (4, 16). These studies found particle deposition to be less in elastase-treated rodents compared with healthy rodents, unlike what was found in the current study. Sweeney et al. (16) found less deposition of 0.45-μm-diameter particles in spontaneously breathing elastase-treated hamsters than in healthy control hamsters. Three factors may explain the differences between the two studies: emphysema-like morphology severity, breathing patterns, and particle size. First, it is likely that the hamsters had developed a more severe form of emphysema than the rats used in the current study because hamsters are more sensitive to elastase treatment than rats (2). The more severe form of emphysema may have led to more alveolar tissue destruction and less available surface area for particles to deposit on, resulting in lower particle deposition compared with the current study. Second, unlike in the current study, hamsters were freely breathing through their nose. As the tidal volume and the breathing frequency were not reported, it is difficult to assess how similar or different these patterns were between the elastase-treated and healthy groups. Also, deposition in the nose is highly dependent upon flow rate. Thus, if inhaled flow rates were higher in spontaneously breathing elastase-treated animals than in healthy rats, nasal deposition would be higher in the elastase-treated animals and a lower number of particles would be able to penetrate in the lung and deposit. Third, differences in deposition may be attributed to the differences in particle size [0.45 (16) vs. 1.22 μm].

In another study, Damon et al. (4) found less deposition in elastase-treated rats compared with healthy rats for nose-only free-breathing exposure of particles with MMAD of 2 ± 1.84 μm. As in study of Sweeney et al. (16), no data on respiratory parameters were reported, and therefore, it is unknown how breathing patterns differed between the healthy and elastase-treated groups. As discussed above, the potential differences in breathing patterns and the different particle sizes may have contributed to the different results found in the two studies.

Finally, while no aerosol deposition study in animals have been performed for controlled breathing conditions, a study of aerosol deposition in human subjects during controlled breathing showed that deposition of 1-μm-diameter particles was significantly higher in patients with COPD than in normal subjects (7), observations that are in agreement with this study.

### Study Limitations

Parameswaran et al. (14) showed that L_M_ may not be the most reliable measure of emphysema, especially if the disease is in its early stages and/or heterogeneously distributed in the lung. They showed that defining alveolar space with a parameter that incorporates the mean equivalent diameter of the air spaces, the SD of equivalent diameters, and the skewness of their distribution might be better suited to characterize heterogeneous emphysema. In the current study, although the mean linear intercept, L_m_, tended to be higher in the elastase-treated rats than in the healthy controls, this increase was not statistically significant. To assess the heterogeneity of the disease, the SD and RD of L_M_ was calculated. No significant difference in SD or RD was found between the healthy and elastase-treated rats. However, although the morphometric measurements were not significantly different between healthy and elastase-treated animals, the significant difference in airway pressure, MR signal decay rate, R_2_^*^, and RD of R_2_^*^ between the two groups were all indicative of the presence of emphysema-like morphology. While not performed within this study, a full characterization of the disease distribution within the lobes could have been performed with high-resolution CT or MRI scans.

It is possible that the degassing of the lungs prior to imaging was not as efficient in the elastase-treated rats because of regions of trapped gas. As R_2_^*^ is greatly influenced by the presence of air bubbles (air causes a rapid decay in MR signal), it is possible that the high R_2_^*^ values in the elastase-treated rats were caused by the presence of microscopic bubbles; however, we were unable to specifically measure this. This could explain the significantly higher R_2_^*^ measured in the elastase-treated control rats than in the healthy control rats. As it is reasonable to assume that the effect of microscopic bubbles on R_2_^*^ was similar in both the aerosol-exposed and control elastase-treated groups, the increase in R_2_^*^ between control and aerosol-exposed elastase-treated animals should be reflective of deposited particles. Therefore, the presence of these microscopic bubbles should not significantly affect our findings.

Finally, while it is possible that some particles were translocated into the blood stream upon inhalation, there currently is little evidence that this happens in a significant way for micro particles within the time frame of these experiments. Additionally, we acknowledge that increased inflammatory cell and mucin production, while not measured in this study, may also contribute to the difference in deposition that was observed between the healthy and elastase-treated group.

In summary, this work is the first to study the differences in particle deposition between healthy and elastase-treated rats that were exposed to aerosol particles in a controlled fashion. Data suggest that, in the elastase-treated animals, the emphysema-like morphology was not uniformly distributed among the five lobes of the lung. Nonetheless, particle deposition was higher and more heterogeneously distributed in the elastase-treated lungs than in the healthy lungs. Overall differences in deposition between elastase-treated and healthy groups are likely due to the morphometric changes induced by the elastase treatment, as both aerosol exposure and breathing parameters were kept similar between the two groups. These results suggest that emphysema-like morphological changes to the pulmonary tissue may lead to enhanced deposition of toxic particles that result from cigarette smoke, air pollution, or man-made micrometer particles. This may cause continued destruction of the pulmonary tissue. On the other hand, aerosolized pharmaceuticals that are used in the treatment of respiratory diseases or to be delivered to the body systemically may be enhanced in emphysema.

## GRANTS

The study was funded by National Heart, Lung, and Blood Institute Grant 1R21-HL-087805-02, Burroughs Wellcome Fund at the Scientific Interface, and a National Science Foundation Graduate Fellowship (to J. M. Oakes). Images for the morphometric analysis were collected at the University of California, San Diego School of Medicine Light Microscopy Facility (Grant P30-NS-047101) with the assistance of Jennifer Meerloo.

## DISCLOSURES

No conflicts of interest, financial or otherwise, are declared by the author(s).

## AUTHOR CONTRIBUTIONS

Author contributions: J.M.O., E.C.B., M.S., and C.D. performed experiments; J.M.O. and G.S.T. analyzed data; J.M.O., E.C.B., M.S., and C.D. interpreted results of experiments; J.M.O. prepared figures; J.M.O. drafted manuscript; J.M.O., E.C.B., M.S., and C.D. edited and revised manuscript; J.M.O., E.C.B., M.S., and C.D. approved final version of manuscript; C.D. conception and design of research.
